# Prehabilitation and acute postoperative physical activity in patients undergoing radical prostatectomy: a secondary analysis from an RCT

**DOI:** 10.1186/s40798-019-0191-2

**Published:** 2019-05-22

**Authors:** Darren Au, Andrew G. Matthew, Paty Lopez, William J. Hilton, Rashami Awasthi, Guillaume Bousquet-Dion, Karim Ladha, Franco Carli, Daniel Santa Mina

**Affiliations:** 1 Faculty of Kinesiology and Physical Education, University of Toronto, Toronto, Ontario Canada; 2 University Health Network, Toronto, Ontario Canada; 3 Faculty of Medicine, University of Toronto, Toronto, Ontario Canada; 4 Department of Anesthesia, McGill University Health Centre, Montreal, Quebec Canada; 5 Department of Surgery, Division of Urology, McGill University, Montreal, Quebec Canada; 6 Department of Anesthesia, Faculty of Medicine, McGill University, Montreal, Quebec Canada

**Keywords:** Surgery, Physical activity, Prehabilitation, Prostate cancer, Radical prostatectomy, Accelerometry

## Abstract

**Background:**

Physical activity via early mobilization after surgery is recommended to help reduce the risk of postoperative adverse effects and to improve recovery. We explored whether prehabilitation is associated with differences in physical activity during the postoperative inpatient stay and the week after discharge in men undergoing abdominal surgery.

**Methods:**

This study was a pre-planned secondary analysis of a larger randomized controlled trial of home-based exercise prehabilitation versus control for men undergoing radical prostatectomy. Twenty-one participants in both the prehabilitation and control groups wore accelerometers from postoperative day 1 until 7 days after discharge. Mean physical activity (minutes) during postoperative day 1 (inpatient) and 1 week following hospital discharge (outpatient) were estimated using ANCOVA. Pearson’s correlation coefficients were conducted for mean in- an outpatient physical activity with length of stay and changes in 6-min walk test (6MWT) over the course of the prehabilitation period.

**Results:**

Nineteen participants in each group provided usable accelerometry data for analysis. Inpatient physical activity of light or greater activity during postoperative day 1 for prehabilitation and control groups were 442.5 ± 40.2 and 324.0 ± 40.2 min, respectively (∆ = 117.5 ± 57.8 min, 95%CI [0.04, 235.0]). During the outpatient period, mean daily physical activity was 448.4 ± 31.2 and 491.42 ± 31.2 min for prehabilitation and control participants, respectively (∆ = 42.6 ± 44.9 min; 95% CI [− 134.0, 48.7]). There were no correlations between in- or outpatient physical activity and preoperative changes in 6MWT or length of stay.

**Conclusions:**

Accelerometry-based measurement of physical activity in the acute postoperative period is feasible in older men undergoing abdominal surgery. Prehabilitation may be associated with increased inpatient physical activity; however, larger and longer studies are needed to elucidate any associated effects on clinical and patient outcomes.

**Trial registration:**

Clinicaltrials.gov: NCT02036684 registered January 15, 2014.

**Electronic supplementary material:**

The online version of this article (10.1186/s40798-019-0191-2) contains supplementary material, which is available to authorized users.

## Key points

- Prehabilitation may improve acute postoperative physical activity levels
- Use of wrist-worn accelerometers are feasible in men undergoing abdominal surgery
- Larger longitudinal studies are needed to determine the effect of prehabilitation on postoperative physical activity as it relates to clinical and functional recovery.

## Introduction

Major abdominal surgery is associated with reduced physical activity, a decline in physical function, and psychological distress that can negatively impact recovery [1]. Enhanced recovery after surgery (ERAS) protocols were conceptually introduced in the late 1990s and have become a common feature of the surgical experience to reduce surgical risk and expedite discharge and recovery [2]. ERAS is broadly described as multimodal care performed by a variety of health professionals throughout the span of the patient’s journey, from pre-admission to acute postoperative management [2]. Early mobilization and ambulation after surgery are common to ERAS programs because they have shown to be associated with reduced postoperative complications, length of stay, and overall recovery time that can yield healthcare cost savings without compromising patient well-being [3–5].

While ‘activity as tolerated’ is commonly advised after surgery and ambulation (assisted or unassisted) is often a requirement for discharge, a vast majority of patients are in fact quite sedentary until discharge [6]. Measurement of physical activity during the postoperative, in-hospital period has evolved with the emergence of accelerometry-based activity monitors that can objectively capture patient movement with reasonable reliability and validity [7]. For example, postoperative inpatient step counts have been shown to be negatively correlated with length of stay and predictive of readmission in patients undergoing cardiac surgery [8, 9], and risk for 30- and 60-day readmission following metastatic cancer surgery [10]. Activity during the acute period after discharge has also shown to be a predictor of 30-day hospital readmission in older adults following admission for acute medical illness [11]. Those that were readmitted were found to be walking one third less than those that were not readmitted [11].

In addition to evidence for early ambulation and postoperative rehabilitation in facilitating surgical recovery, a growing body of literature describes the importance of preoperative physical fitness levels for surgical outcomes [12]. Poor preoperative physical fitness has been associated with increased length of stay, increased postoperative complications, longer recovery, and all-cause mortality [12, 13]. Given the importance of physical fitness, and the potential to optimize these factors during the surgical wait time, interest in prehabilitation has grown as a strategy to reduce surgical risk and improve postoperative recovery. Prehabilitation is defined as the process of enhancing the functional and mental capacity of an individual to buffer against potential adverse effects of an impending and significant stressor, such as surgery [14]. Recent reviews of prehabilitation demonstrate that inspiratory muscle training, aerobic, and/or resistance training may reduce postoperative complications, reduce length of stay, and increase physical function [15]. Contemporary prehabilitation interventions have since adopted a multimodal approach that includes elements of nutritional, psychological, and/or exercise. Despite the promising benefit of prehabilitation, the quality of evidence is limited by significant heterogeneity of preoperative interventions, cancer type, and treatment techniques, as well as perioperative care. Furthermore, detailed information regarding effective doses, duration, and adherence is lacking.

The application of ERAS preoperative protocols are aimed to reduce physiological and psychological stress through education and counseling as well as nutritional optimization; however, the inclusion of exercise has yet to be incorporated. The potential impact prehabilitation on acute postoperative physical activity or ambulation that is commonly a part of ERAS protocols has not been assessed for the abovementioned outcomes. Increases in acute postoperative physical activity that result from improved preoperative fitness levels or education on the benefits of exercise may, in part, explain some of the prehabilitation benefits. To examine the relationship between prehabilitation and acute postoperative physical activity levels, we compared physical activity levels to 1 week after surgery in patients who underwent radical prostatectomy who were randomized to either prehabilitation or control.

## Methods

This study is a secondary analysis from data gathered during a multi-centered, randomized controlled trial that assessed the feasibility of home-based prehabilitation (prehab) versus control in men undergoing radical prostatectomy (clinicaltrial.gov: NCT02036684, 16, 17]**.** The study protocol is published elsewhere [16] and is briefly described below. Ethics approval for the main trial (including data related to the secondary analysis) was obtained and the study was performed in accordance with the standards of ethics outlined in the Declaration of Helsinki.

Eighty-six (*n* = 86) participants were recruited from February 2014 to September 2015 in the primary study and randomized to prehab or control. The prehab intervention entailed individualized, home-based, moderate intensity aerobic, and resistance exercises prescribed and demonstrated shortly after consenting to surgery. Participants were provided with exercise bands, exercise mat, and a stability ball to complete their program, in addition to a manual detailing their exercise prescription with supporting behavior change strategies. In addition to the general conditioning exercise prescription, prehab participants received information and coaching on pelvic floor exercises to complete prior to surgery targeting earlier recovery of urinary control after surgery. The control group received the same pelvic floor exercise training regimen as the prehab group and were given a book on maintaining a healthy lifestyle after a prostate cancer diagnosis with no further exercise support. Both groups were asked to maintain their prescribed activities until their date of surgery (typically 4–8 weeks), but not specifically instructed to resume their respective activities after surgery.

Wrist-worn accelerometers (Actiwatch 2, Philips Healthcare, Respironics, USA) were provided shortly after surgery (either in the post-anesthetic care unit or upon admission to the ward) with instructions to wear the device for at least 1 week after discharge. While the devices are waterproof, instructions did not specify the requirement to keep the device on or to remove during bathing activities. A recent validation study for the Actiwatch 2 has shown the device to be highly comparable to indirect calorimetry and specifically has demonstrated to be able to distinguish between sedentary and moderate-to-vigorous physical activity classifications in the healthy population [17]. The Actiwatch has not been previously validated in our population; however, the use of hip-worn accelerometers has been shown to be tolerable and reasonably consistent with activity diaries in capturing postoperative physical activity in patients undergoing abdominal surgery [6]. Inpatient physical activity was captured over a 24-h period starting at 8 am of postoperative day 1. Inpatient physical activity was limited to a 24-h period because discharge was typically on postoperative day 2 (38.3 ± 16.6 h). Outpatient physical activity was collected over 7 days, starting at 8 am of the first day after discharge.

Similar to the protocol described in National Health and Nutrition Examination Survey (NHANES) [18], device-derived acceleration data were converted to activity counts based on the manufacturer’s algorithm and activity counts were summed to yield counts per minute (cpm; i.e., activity counts in 60-s epoch). A cpm of less than 100 was categorized as ‘sedentary’ behavior [18]. The total daily physical activity volume was thus considered to be the time (minutes) spent at 100 cpm or greater. To account for periods of time that participants may have removed the device, only those participants with 10 or more hours of wear-time daily were included in the analysis [19, 20]. Non-wear time was defined as zero activity for 60 consecutive minutes [19, 20].

Baseline demographics and clinical characteristics were summarized using descriptive statistics for continuous and categorical data. Demographic and clinical characteristic differences between groups were conducted using *t* test and Fisher’s exact test where appropriate. Analysis of covariance (ANCOVA) was used to estimate mean physical activity and group differences on postoperative day 1 and 1 week following hospital discharge. This model was adjusted for age, wait time, and length of stay. To assess the relationship between changes in physical fitness during the pre-operative period and postoperative physical activity, Pearson’s correlation coefficients were calculated for changes in 6MWT from baseline to pre-surgery and both the mean in- and outpatient physical activity volume for all patients. Pearson’s correlations were similarly conducted to assess the relationship between mean postoperative in- and outpatient physical activity and length of stay. All statistical analyses were done on R version 3.4.4. and alpha was set to 0.05.

## Results

Forty-two of the *n* = 86 participants were provided with accelerometers (due to a limited number of accelerometers) and all were returned in functional order. Ninety percent (*n* = 38/42; prehab: *n* = 19; control: *n* = 19) of participants that received the accelerometer provided usable data for the analyses (i.e., greater than 10 h of wear-time each day). Patient baseline demographics and disease characteristics are shown in Table 1 and were comparable between groups. Furthermore, the sub-sample of participants included in this analysis shared comparable demographic and disease characteristics to those that did not receive accelerometers (Additional file 1: Table S1). Estimated mean physical activity and group differences are shown in Table 2. Mean inpatient physical activity minutes were 442.5 ± 40.2 and 324.0 ± 40.2 in prehab and controls, respectively (∆ = 117.5 ± 57.8 min, 95% CI: [0.04, 235.0], *p* < 0.05). Mean daily physical activity during the week after discharge was 448.4 ± 31.2 and 491.4 ± 31.2 min in prehab and controls, respectively (∆ = 42.6 ± 44.9 min; 95% CI [− 134.0, 48.7]). There were no statistically significant correlations between mean 6MWT change or length of stay with mean in- and outpatient physical activity minutes (Table 3).

**Table 1:** Patient demographics and clinical characteristics

Demographic characteristics	Prehab (*n* = 19)	Control (*n* = 19)	*p* value
Age (years), mean ± SD	61.4 ± 7.8	58.4 ± 6.1	.203
BMI (kg/m^2^), mean ± SD	26.4 ± 4.6	25.6 ± 4.9	.598
Ethnicity [*n* (%)]			
White/Caucasian	13 (68.4)	14 (73.7)	.378
Black/Afro-Caribbean/African	3 (15.8)	2 (10.5)	
Ashkenazi Jewish	1 (5.3)	0	
East and South Asian	1 (5.3)	2 (10.5)	
Arabic	0	1 (5.3)	
Hispanic	1 (5.3)	0	
Annual income [*n* (%)]			
Less than $40,000	5 (26.3)	5 (26.3)	.311
$40,000–$80,000	8 (42.1)	4 (21.1)	
More than $80,000	6 (31.6)	10 (52.6)	
Marital status [*n* (%)]			
Married (including common law)	12 (63.2)	12 (63.2)	.673
Divorced	4 (21.1)	3 (15.8)	
Single (never married)	2 (10.5)	3 (15.8)	
Separated	0	1 (5.3)	
Widowed	1 (5.3)	0	
Education [*n* (%)]			
Less than high school	3 (15.8)	0	.345
High school graduate	3 (15.8)	4 (21.1)	
Community college	5 (26.3)	4 (21.1)	
University undergraduate or graduate degree	8 (42.1)	10 (52.6)	
Other	0	1 (5.3)	
Working status [*n* (%)]			
Full-time	7 (36.8)	10 (52.6)	.086
Unemployed	0	1 (5.3)	
Part-time	2 (10.5)	5 (26.3)	
Retired	10 (52.6)	3 (15.8)	
Smoking [*n* (%)]			
No	18 (94.7)	18 (94.7)	1.00
Yes	1 (5.3)	1 (5.3)	
Disease and treatment information			
Wait time (days), mean ± SD	83.8 ± 21.2	92.1 ± 34.3	.378
Cancer T stage [*n* (%)]			
T1	0	1 (5.3)	.201
T2	7 (36.8)	11 (57.9)	
T3	12 (63.2)	7 (36.8)	
Gleason score [*n* (%)]			
7	17 (89.5)	16 (84.2)	.307
8	0	2 (10.5)	
10	2 (10.5)	1 (5.3)	
Surgical approach [*n* (%)]			
Robot-assisted	14 (73.7)	16 (84.2)	.691
Open	5 (26.3)	3 (15.8)	
Length of stay (h), mean ± SD	38.7 ± 18.1	38.0 ± 15.4	.893
Days of catheterization (days), mean ± SD	13.8 ± 2.4	13.0 ± 3.6	.401
Accelerometer measurement			
Daily accelerometer wear time (h), mean ± SD	23.3 ± 0.8	23.6 ± 0.6	.233

*BMI* body mass index

**Table 2:** Mean estimated physical activity and group differences during postoperative day 1 and 1 week following discharge

Group	Mean ± SE	95% CI	∆ Prehab–control Mean ± SE	95% CI
Mean physical activity (min) during postoperative day 1				
Control	324.94 ± 40.23	(243.1, 406.8)	117.53 ± 57.75	(0.04, 235.03)
Prehab	442.48 ± 40.23	(360.6, 524.3)		
Mean daily physical activity (min) during post-discharge week 1				
control	491.42 ± 31.2	(427.4, 512.1)	− 42.6 ± 44.9	(−134.0, 48.7)
prehab	448.4 ± 31.2	(384.8, 512.1)

**Table 3:** Pearson’s correlation coefficients between change in 6-min walk test and postoperative length of stay with physical activity min

		Time point			
Outcome	Group	Postoperative day 1	*p* value	Total daily physical activity (min) 1 week post-discharge	*p* value
∆ 6MWT	Consolidated	− 0.03	.857	0.02	.883
	Control	− 0.27	.398	− 0.12	.629
	Prehab	0.23	.348	0.17	.494
LoS	Consolidated	− 0.29	.081	− 0.15	.367
	Control	− 0.37	.123	0.13	.584
	Prehab	− 0.26	.278	− 0.42	.072

Pearson *r* are presented with *p* values underneath in parentheses; *6MWT* 6-min walk test, *LoS* length of stay

## Discussion

We analyzed physical activity volume during the postoperative inpatient stay and 1 week following discharge relative to participation in prehabilitation prior to radical prostatectomy. To our knowledge, acute postoperative physical activity has not previously been assessed in this population, nor has it been examined in patients participating in a prehabilitation intervention across surgical populations. In the primary reported study, we demonstrated the feasibility of prehabilitation and showed high adherence rates for the prescribed aerobic and whole-body resistance exercise, 84% and 79%, respectively [21]. In this secondary analysis, we observed that those who participated in prehabilitation had significantly more physical activity during postoperative day 1 compared to control participants; however, both groups were comparable in physical activity during the week following discharge. There were no statistically significant relationships between physical activity over the periods we assessed with either change in functional capacity (6MWT) during the preoperative period or inpatient length of stay.

Early ambulation is a common component in many ERAS protocols, where mobilization may be recommended as early as the evening of surgery and regular ambulation during postoperative day 1 [22]. Preoperatively, the role of prehabilitation within ERAS protocols is only weakly supported, primarily due to the absence of high-quality data among prehabilitation studies with outcomes pertinent to ERAS protocols, such as all-cause mortality, postoperative morbidity, hospital length of stay, and healthcare cost. Linking the two paradigms may prove effective at increasing early postoperative physical activity if it can be planned for with education and activity targets during a prehabilitation period.

### Study limitations

Although the secondary analysis was planned a priori*,* it warrants conservative interpretation in light of several limitations. First, the sub-sample of participants with activity monitors was small and insufficient to draw strong conclusions on the effect of prehabilitation on postoperative physical activity. Second, radical prostatectomy may not be the most optimal surgical procedure to understand the potential relationship between physical activity and health outcomes given that it is relatively low-risk and most patients were discharged on postoperative day 2. Furthermore, radical prostatectomy requires postoperative catheterization postoperatively until 10–14 days after surgery that may be the most significant predictor of physical activity undermining its potential benefits. Third, construct validity of postoperative physical activity was not assessed via diaries or direct observation to confirm wear time or ambulatory physical activity (however, as noted previously, data were only included from participants whose wear time met the NHANES criterion). Finally, the intervention was not specifically designed to encourage acute post-operative physical activity, nor did it include psychological or nutritional prehabilitation that may have impacted patient activity levels. In light of a growing body of literature supporting multimodal prehabilitation, future studies should consider comprehensive prehabilitation strategies that emphasize and support early ambulation and physical activity following surgery.

## Conclusion

The use of accelerometry to measure postoperative physical activity in the inpatient, and acute outpatient setting is feasible in older men undergoing abdominal surgery. Prehabilitation may play a role in increasing postoperative inpatient physical activity; however, more research with larger sample sizes is needed to evaluate the potential effect preoperative interventions that specifically target these behaviors.

## Additional file

Additional file 1: **Table S1.** Participant demographics and clinical characteristics between those that were provided with accelerometers and those that were not. (DOCX 18 kb)

## Abbreviations

6MWT: 6-min walk test
ANCOVA: Analysis of covariance
cpm: Counts per minute
ERAS: Enhanced Recovery After Surgery
NHANE: National Health and Nutrition Examination Survey
Prehab: Prehabilitation
