# Supplementary material for: Prehabilitation and acute postoperative physical activity in patients undergoing radical prostatectomy: a secondary analysis from an RCT
Source: Sports Med Open. 2019 May 22;5:18. doi: 10.1186/s40798-019-0191-2 (PMC6531507; doi:10.1186/s40798-019-0191-2)
Supplement: Supplementary file 1 — Table S1. Participant demographics and clinical characteristics between those that were provided with accelerometers and those that were not. (DOCX 18 kb) [file 40798_2019_191_MOESM1_ESM.docx]

Supplementary Table 1. Participant demographics and clinical characteristics between those that were provided with accelerometers and those that were not.

| **Demographic Characteristics** | **No accelerometer (n=44)** | **Accelerometer (n=42)** | ***p*-value** |
| --- | --- | --- | --- |
| *Age* (years), mean±SD | 63.0±7.6 | 60.3±7.25 | .101 |
| *BMI* (kg/m^2^), mean±SD | 27.3±4.5 | 26.8±4.0 | .617 |
| *Ethnicity* [n (%)] |  |  |  |
| White/Caucasian | 30 (68.2) | 30 (71.4) | .926 |
| Black/Afro-Caribbean/African | 7 (15.9) | 4 (9.5) |  |
| Ashkenazi Jewish | 0 | 1 (2.4) |  |
| East and South Asian | 0 | 4 (9.5) |  |
| Arabic | 0 | 2 (4.8) |  |
| Hispanic | 0 | 1 (2.4) |  |
| South East Asian | 1 (2.3) | 0 |  |
| Other | 5 (11.4) | 0 |  |
| Missing | 1 (2.3) | 0 |  |
| *Annual Income* [n (%)] |  |  |  |
| Less than $40,000 | 13 (29.5) | 10 (23.8) | .056 |
| $40,000 - $80,000 | 21 (47.7) | 16 (38.1) |  |
| More than $80,000 | 7 (15.9) | 16 (38.1) |  |
| Missing | 3 (6.8) | 0 |  |
| *Marital Status* [n (%)] |  |  |  |
| Married (including common law) | 35 (79.5) | 28 (66.7) | .232 |
| Divorced | 4 (9.1) | 5 (11.9) |  |
| Single (never married) | 4 (9.1) | 4 (9.5) |  |
| Separated | 0 | 3 (7.1) |  |
| Widowed | 0 | 2 (4.8) |  |
| Missing | 1 (2.3) | 0 |  |
| *Education* [n (%)] |  |  |  |
| Less than high school | 6 (13.6) | 1 (2.4) | .136 |
| High school graduate | 8 (18.2) | 10 (23.8) |  |
| Community college | 6 (13.6) | 9 (21.4) |  |
| University undergraduate or graduate degree | 18 (40.9) | 21 (50.0) |  |
| Other | 4 (9.1) | 1 (2.4) |  |
| Missing | 2 (4.5) | 0 (0.0) |  |
| *Working Status* [n (%)] |  |  |  |
| Full-time | 17 (38.6) | 18 (42.9) | .325 |
| Unemployed | 2 (4.5) | 1 ( 2.4) |  |
| Part-time | 6 (13.6) | 11 (26.2) |  |
| Retired | 17 (38.6) | 12 (28.6) |  |
| Missing | 2 (4.5) | 0 |  |
| *Smoking* [n (%)] |  |  |  |
| No | 40 (90.9) | 40 (95.2) | .562 |
| Yes | 3 (6.8) | 2 (4.8) |  |
| Missing | 1 (2.3) | 0 (0.0) |  |
| **Disease and Treatment Information** |  | |  |
| *Wait time (days)*, mean±SD | 31.4±16.93 | 30.6±17.01 | .820 |
| *Cancer T Stage* [n (%)] |  |  |  |
| Benign | 1 (2.3) | 0 |  |
| T1 | 0 | 1 (2.4) | .229 |
| T2 | 15 (34.1) | 19 (45.2) |  |
| T3 | 25 (56.8) | 22 (52.4) |  |
| Missing | 3 (6.8) | 0 |  |
| *Gleason Score* [n (%)] |  |  |  |
| Undistinguished | 1 (2.3) | 0 |  |
| 7 | 25 (58.1) | 36 (85.7) | .058 |
| 8 | 4 (9.3) | 2 (4.8) |  |
| 9 | 7 (16.3) | 1 (2.4) |  |
| 10 | 6 (14.0) | 3 (7.1) |  |
| *Surgical Approach* [n (%)] |  |  |  |
| No surgery | 4 (9.1) | 0 | .134 |
| Robot-assisted | 32 (72.7) | 34 (81.0) |  |
| Open | 8 (18.2) | 8 (19.0) |  |
| *Length of stay (hours)*, mean±SD | 25.6±17.8 | 25.6±13.7 | 0.994 |
| BMI: body mass index.  Comparison for ethnicity was white compared to all others. | | | |
